# Mobilization of Transplanted Bone Marrow Mesenchymal Stem Cells by Erythropoietin Facilitates the Reconstruction of Segmental Bone Defect

**DOI:** 10.1155/2019/5750967

**Published:** 2019-04-01

**Authors:** Jun Li, Zeyu Huang, Bohua Li, Zhengdong Zhang, Lei Liu

**Affiliations:** Department of Orthopedics, West China Hospital, Sichuan University, 37# Wainan Guoxue Road, Chengdu 610041, China

## Abstract

Reconstruction of segmental bone defects poses a tremendous challenge for both orthopedic clinicians and scientists, since bone rehabilitation is requisite substantially and may be beyond the capacity of self-healing. Bone marrow mesenchymal stem cells (BMSCs) have been identified as an optimal progenitor cell source to facilitate bone repair since they have a higher ability for proliferation and are more easily accessible than mature osteoblastic cells. In spite of the potential of BMSCs in regeneration medicine, particularly for bone reconstruction, noteworthy limitations still remain in previous application of BMSCs, including the amount of cells that could be recruited, the compromised bone migration of grafted cells, reduced proliferation and osteoblastic differentiation ability, and likely tumorigenesis. Our current work demonstrates that BMSCs transplanted through the caudal vein can be mobilized by erythropoietin (EPO) to the bone defect area and participate in regeneration of new bone. Based on the histological analysis and micro-CT findings of this study, EPO can dramatically promote the effects on the osteogenesis and angiogenesis efficiency of BMSCs in vivo. Animals that underwent EPO+BMSC administration demonstrated a remarkable increase in new bone formation, tissue structure organization, new vessel density, callus formation, and bone mineral density (BMD) compared with the BMSCs alone and control groups. At the biomechanical level, we demonstrated that combing transplantation of EPO and BMSCs enhances bone defect reconstruction by increasing the strength of the diaphysis, making it less fragile. Therefore, combination therapy using EPO infusion and BMSC transplantation may be a new therapeutic strategy for the reconstruction of segmental bone defect.

## 1. Introduction

Segmental bone defects often arise from trauma, tumor resection, congenital malformation, skeletal diseases, and aseptic loosening around implants. Reconstruction of segmental bone defects poses a tremendous challenge for both orthopedic clinicians and scientists, since bone rehabilitation is requisite substantially and is incapable of self-healing [1]. Although many methods for bone reconstruction have been established, such as autologous bone grafts, allografts, bone substitute, and growth factor delivery, they all have specific indications and limitations [2–4]. Autologous bone grafts exhibit high rates of healing without immunogenicity, yet this approach is associated with donor site morbidity, chronic pain, restricted grafting material, and compromised bone mass in patients with osteoporosis [5]. Allografts resolve some of these donor issues but are further complicated by a higher risk of nonunion, recipient infection, host immune responses, and poor revascularization [6, 7].

In the past decades, numerous tissue engineering approaches, such as synthetic bone graft substitutes, free vascularized grafts, and growth factors, have been investigated for their therapeutic potential in reconstruction of segmental bone defects. However, these approaches are still more complicated and more expensive when compared to conventional bone grafting [8]. As an alternative bone regeneration strategy, stem cell-based regenerative medicine approaches are being explored to address the problems associated with current bone grafting surgeries [9].

Bone marrow mesenchymal stem cells (BMSCs) have been identified as an optimal progenitor cell source to facilitate bone repair since they have a higher proliferation ability and are more easily accessible than mature osteoblastic cells [10]. BMSCs possess multidirectional potential and can differentiate into osteoblasts, adipocytes, chondrocytes, tendon cells, neurons, and myoblasts [11]. It has been demonstrated in clinical studies that BMSCs are efficient in the treatment of nonunion fracture and bone defect and reinforce the effect of distraction osteogenesis [12, 13]. Under some circumstances, however, there are limitations with the accessibility of eligible bone marrow due to aging and diseases of the patients [14]. Autologous BMSC grafting is a potential strategy to facilitate bone regeneration, yet this strategy may be ineffective for patients suffering from osteoporosis and arthritis due to the reduction of proliferation and osteoblastic differentiation ability of BMSCs [15]. Other studies confirmed that BMSCs derived from patients with osteopenia had reduced osteogenic differentiation capability, which could lead to delayed union or nonunion [16–18].

Previously, we reported that grafted BMSCs can be recruited by erythropoietin (EPO) to the sites of injured spinal cord and promote functional recovery [19]. It has also been demonstrated that EPO contributes to the transition of transplanted BMSCs to target tissues, incorporating the acute injured kidney [20], calvarial defect [21] area, and infarcted heart [22]. This strategy has been verified to be sterile, safe, and efficient. It is then well founded to hypothesize that transplanted BMSCs can be mobilized by EPO to the bone defect area and enhance the bone defect reconstruction without great concerns for safety. Stromal cell-derived factor (SDF) 1 and its receptor CXCR4 play essential roles in regulating mobilization and migration of BMSCs to the injury sites [19, 23]. The homing of BMSCs to the ischemic myocardium was significantly blocked when the SDF1/CXCR4 axis was inhibited [24]. The aim of this study was therefore to (a) verify the feasibility of using EPO to recruit transplanted BMSCs toward the bone defect site, (b) evaluate the effect of this combination strategy for bone defect healing, and (c) investigate the potential role of the SDF-1/CXCR4 pathway in regulating the migration and recruitment of transplanted BMSCs to the bone defect area.

## 2. Materials and Methods

### 2.1. Derivation, Culture, and Labeling of BMSCs

BMSCs were harvested from female Sprague-Dawley (SD) rats. Briefly, the rats were decapitated after anesthesia using 10% chloral hydrate. Femurs and tibiae were aseptically removed, and bone marrow was flushed with phosphate-buffered saline (PBS) via a sterile syringe. Bone marrow samples were centrifuged for 5 minutes at 1000g and then placed in Dulbecco's modified Eagle's medium (DMEM, Invitrogen, US) and replenished with 10% fetal bovine serum (FBS, Beyotime, China). The growth medium was changed every 3 days. BMSCs were passaged until 80-90% confluence was attained. To trace the transferring of grafted BMSCs to the bone defect area in vivo, plasmid transfection of recombinant adenovirus encoding green fluorescent protein (Ad-GFP) was carried out referring to the instruction of the manufacturer.

### 2.2. Animals

120 adult female SD rats, with weight of 250-300 g and age of approximately 12 weeks, were used in this research. All animal experimental protocols were reviewed and authorized by the Ethics Committee for Animal Experiments of the West China School of Medicine, Sichuan University, China. The rats were kept in an indoor facility for one week before the beginning of the experiments, with accessible food and water in conditions of 21°C, 60% atmospheric humidity, and a 12 h light/dark cycle.

### 2.3. Immunocytochemistry Assay

The slides of passage 3 BMSCs were fixed with 4% paraformaldehyde (Sinopharm, China) for 15 min and then rinsed 3 times using PBS. Afterwards, the cell slides were ventilated with 0.5% Triton X-100 (Beyotime, China) at room temperature for 20 min and blocked with goat serum for 30 min. A PBS wash followed each step. Diluted primary antibodies were added to the slides and coincubated at 4°C overnight. The primary antibodies used for immunostaining included mouse anti-CD90 (1/500, Abcam), mouse anti-CD11b (1/200, Abcam), mouse anti-CD44 (1/500, Abcam), mouse anti-CD29 (1/200, Abcam), rabbit anti-CD45 (1/100, Abcam), and rabbit anti-CD271 (1/500, Abcam). After 3 rinses with PBS, the slides were coincubated with diluted secondary primary antibodies (DyLight® 488-goat anti-rat IgG, 1/100, Invitrogen; DyLight® 488-goat anti-rabbit IgG, 1/100, Invitrogen) for 1 h. Following 3 times of PBS wash, the slides were coincubated with 4′,6-diamidino-2-phenylindole (DAPI) avoiding light for 5 min. After 4 subsequent PBS washes, the slides were dried using absorbent paper and mounted with an antifluorescence quenching agent (Southern Biotech, Alabama, US). Images were collected using a fluorescence microscope (Olympus BX53, Japan).

### 2.4. Multilineage Differentiation Assay

The multipotency of BMSCs was detected by their differentiation into osteocytes and adipocytes in vitro [25]. The adipogenic differentiation was assessed by oil red O staining. The induction medium was composed of 10% fetal bovine serum (FBS, Beyotime, China), 0.5 mM 3-isobutyl-1-methylxanthine (IBMX; New Jersey, US), 10 mg/l insulin, 50 *μ*M indomethacin, and 0.1 *μ*mol/l dexamethasone. The medium was substituted every 3 days, and the cells were dyed via oil red O solution and observed using a light microscope at the 14th day. Osteogenic differentiation was evaluated by alkaline phosphatase (ALP) staining. The inductive medium was composed of 10 mmol/l sodium *β*-glycerophosphate, 10% FBS, 50 *μ*mol/l ascorbic acid, and 0.25 *μ*mol/l dexamethasone. The medium was changed every 3 days, and the cells were dyed via ALP solution and observed at the 21st day.

### 2.5. Western Blot Assay

The passage 3 BMSCs were adjusted to a density of 1.0 × 10^6^/ml and inoculated in a 6-hole plate. DMEM containing 2% FBS was added. Cells were cultured until 80-90% confluence was achieved. BMSCs were then coincubated with gradient concentration of EPO (0 IU/ml, 50 IU/ml, 100 IU/ml, and 150 IU/ml) for 48 h, 3 holes for each concentration. Cells were collected and synchronized with 400 *μ*l cell lysis buffer for 30 min and cooked in boiling water for 10 min. Afterwards, cellular debris and cell lysis buffer were centrifuged at 8000g for 5 min. The supernatants were collected for sodium dodecyl sulfate polyacrylamide gel electrophoresis (AMRESCO, US) and transferred to polyvinylidene fluoride (PVDF; Millipore, US) membrane. After being blocked using Tris-buffered saline Tween 20 (Bioscience, China), the PVDF membranes were hatched with anti-CXCR4 antibody (1 : 1000, Santa Cruz, US) or rabbit anti-GAPDH antibody (1 : 1000, Xianzhi Biological Ltd., China) as primary antibodies at 4°C overnight, followed by incubation with goat anti-rabbit antibody labeled with horseradish peroxidase (1: 50,000, Santa Cruz, US) at 37°C for 2 h. The objective protein was observed with enhanced chemiluminescent detection kit (ECL, Thermo, US) and exposed to X-ray films. The procedure was repeated for five times. The gray value of each band was quantified using BandScan 5.0.

### 2.6. Transwell Migration Assay

The migration assay was conducted via Transwell chambers (Biosciences, New Jersey, US). In the control group, the upper chambers were loaded with 1 × 10^5^ BMSCs in 200 *μ*l of serum-free culture medium and the lower chambers with 800 *μ*l of DMEM. In the experimental group, the upper chambers were hatched with 200 *μ*l serum-free culture medium comprising 1 × 10^5^ BMSCs, and the lower chambers were added with 100 U/ml rhEPO and 10% FBS in 800 *μ*l of DMEM. For the chemotaxis inhibition group, the upper chambers were loaded with 1 × 10^5^ BMSCs that were incubated with 10 *μ*g/ml AMD3100 (Pfizer, NY, US) in 200 *μ*l serum-free culture medium at 37°C for 2 h, and the lower chambers were loaded with 800 *μ*l of DMEM containing 100 U/ml rhEPO and 10% FBS. After incubating for 18 h, unmigrated cells in the upper chamber were eliminated and the membranes were immobilized using 4% paraformaldehyde for 30 min. The cells that located to the lower side of the filter were dyed using 5% crystal violet dye solution for 20 min and then washed with PBS and checked using an inverted phase contrast microscope (Olympus X71, Tokyo, Japan). The OD value is calculated using ImageJ. The procedure was repeated for five times.

### 2.7. Construction of Bone Defect Model and Administration of Drugs

Animals were anesthetized via intraperitoneal injection of 10% chloral hydrate. The left hind limb was cleared, sterilized with alcohol, and covered using sterile drapes. A longitudinal incision on the posteromedial tibia was made, and subcutaneous and muscle layers were bluntly dissected. A segmental bone defect with 5 mm length was created with a bone saw and fixed with intramedullary nail in a retrograde manner. The muscle, subcutaneous tissues, and skin were sutured stepwisely. All operating procedures were performed aseptically to avoid potential infection of pathogens. The rats were randomly assigned to four groups (*n* = 16 per group). Rats in the BMSC group were subjected to tail vein injection of 10 *μ*l BMSC suspension containing approximately 3 × 10^4^ cells. In the EPO+BMSC group, besides the vein injection of BMSCs, rhEPO (5 × 10^3^ IU/kg) was administrated via intramuscular injection. In the BMSC+EPO+AMD3100 group, following injection of BMSCs and EPO, the AMD3100 solution (5 mg/kg, Pfizer Compounds, NY, US) was administered with intramuscular injection. In the model control group, 10 *μ*l PBS was injected intramuscularly. The tibias were obtained at 4 and 8 weeks and fixed with 10% formaldehyde for further detection.

### 2.8. Fluorescent Microscopy

To track the migration of transplanted BMSCs in vivo, immunofluorescence assay was conducted at 1, 7, and 28 days after transplantation. Briefly, the specimens of defected bone were frozen and embedded with an optimal cutting temperature compound (OCT, Leica Biosystems, China). Fluorometric analysis was performed by immunofluorescent microscopy to determine the presence and amount of GFP-labeled BMSCs among the defect areas.

### 2.9. Histological and Histomorphometric Analysis

At 4 and 8 weeks after operation, the rats were sacrificed and tibia specimens were individually retrieved. The specimens were immobilized with 10% formalin and decalcified using ethylenediaminetetraacetic acid (Invitrogen, US). The fixed tissues were dehydrated with graded ethanol series (80%–100%) and embedded with paraffin. The embedded specimens were cut into 5 *μ*m sections and dyed using hematoxylin and eosin (HE) or Masson's trichrome. Specimens were inspected through a BX53 microscope (Olympus, Tokyo, Japan). The magnitude of newly generated bone and the number of new blood vessels were detected using Image-Pro Plus 6.0 Software (Meyer Instruments Inc., US). The area of newly formed bone was normalized by the total defect area to generate a percentage of new bone formation. The density of neovascularity was measured as the number of new blood vessels divided by the defect area. Three samples were detected for each group, and 5 sections per sample were used for measurements.

### 2.10. Biomechanical Evaluation

At 4 and 8 weeks after operation, the tibias were extracted for biomechanical testing. The residual soft tissues were removed, and the distal end of the tibia was trimmed at proper length to make the defect the center of the specimens. A three-point flexural test was performed on a biomechanical tester (Ruige Technology, China). The stiffness and ultimate loading were measured to evaluate the biomechanical properties. Three samples were detected in each group. The data are plotted as mean ± SD.

### 2.11. Microcomputed Tomography (CT) Analysis

A Quantum GX micro-CT (PerkinElmer, MA, US) was used to assess the bone volume and bone mineral density across the defective area at 8 weeks. The specimen was placed with its long axis parallel to the scanner's rotation axis. The scanned area is 36 mm, the voxel size resolution is 4.5 *μ*m, and the scanning time is 14 min for each sample. Three-dimensional reconstruction of each specimen was generated to visualize the distribution of mineralized tissue formation within the defective area. Bone volumetric restoration and analysis were conducted and recorded using Analyze 12.0 software provided by PerkinElmer.

### 2.12. Statistical Analysis

Statistical analysis was performed using the Statistical Package for the Social Sciences (SPSS 22.0, IBM, US). All data were expressed as mean value ± standard deviation (SD). Statistical comparisons were conducted using analysis of variance (ANOVA) in which a *P* value of less than 0.05 was considered statistically significant. Tukey's multiple comparison tests were used at a *P* value of 0.05.

## 3. Results

### 3.1. Characterization of BMSCs In Vitro

**Figure 1:**
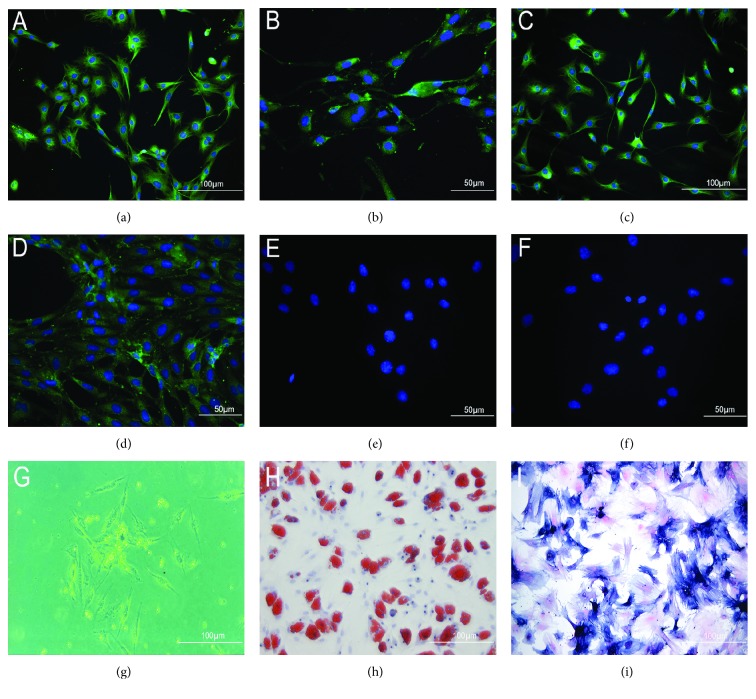
The characteristics of BMSCs. The passage 3 BMSC immunofluorescent labelings were positive for CD29 (a), CD44 (b), CD90 (c), and CD271 (d) and negative for CD11b (e) and CD45 (f). (g) BMSCs manifested typical spindle or polygonous shape and adhered to the plastic culture plate. (h) Positive oil red O staining on day 14 revealed the differentiation of passage 3 BMSCs into adipocytes. (i) ALP staining of passage 3 BMSCs indicated formation of calcification nodes on day 21. Scale bar is 100 *μ*m for (a, c, g, h, and i) and 50 *μ*m for (b, d, e, and f).

BMSCs were characterized according to the following criteria: specific immunophenotype, growth by static adherence, and multipotent differentiation ability. Immunophenotype analysis indicated that the cells harvested were strongly positive for CD29, CD44, CD90, and CD271 (Figures 1(a)–1(d)) and negative for CD11b and CD45 (Figures 1(e) and 1(f)). BMSCs manifested as typical spindle or polygonal shape and adhered to the culture plate (Figure 1(g)). The multipotency of BMSCs was confirmed as positive oil red O staining and positive ALP staining were observed (Figures 1(h) and 1(i)), indicating the differentiation of BMSCs into adipocytes and osteocytes.

### 3.2. EPO Induces BMSC Migration In Vitro

**Figure 2:**
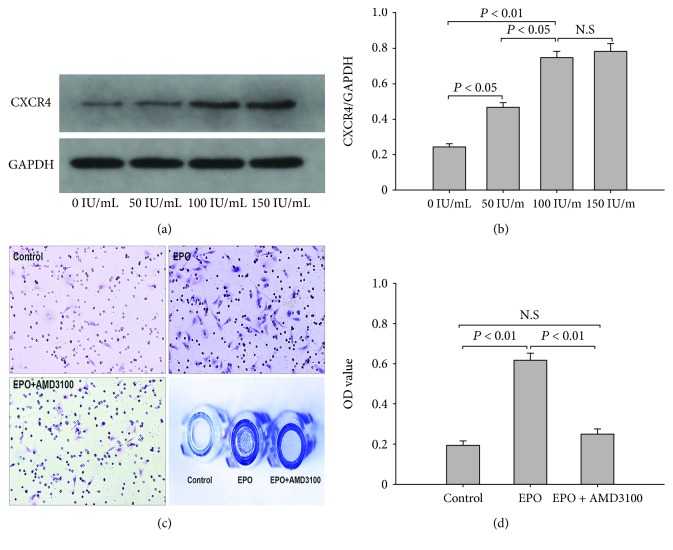
Western blot and Transwell migration assay. (a) Western blot assay revealed that the expression levels of CXCR4 increased with the increment of EPO centration. (b) The CXCR4/GAPDH ratio was the lowest without EPO intervention and the highest at the maximum concentration of EPO. (c) EPO-induced migration of BMSCs was confirmed by inverted phase contrast microscopy. (d) The OD value in the EPO group was significantly higher than that of the control groups, while no significant difference was detected between the EPO+AMD3100 group and the control group. The data are plotted as mean ± SD. N.S=no significant difference.

To study the potential role of EPO intervention on the migration of BMSCs, we performed Western blot and Transwell migration assay to explore the CXCR4 expression levels on BMSCs and migration rates of BMSCs. Our results indicated that after coincubation with the gradient concentration of EPO for 48 h, the expression levels of CXCR4 increased in a dose-dependent manner (Figures 2(a) and 2(b)). The CXCR4/GAPDH ratio was the lowest (0.243 ± 0.017) without EPO intervention and the highest (0.781 ± 0.039) with the maximum concentration of EPO; there was no significant difference when treated with EPO at 100 IU/ml and 150 IU/ml (*P* = 0.3225). In addition, the OD value in the EPO group (0.616 ± 0.038) was significantly higher than that of the control groups (0.193 ± 0.022, *P* < 0.001; Figures 2(c) and 2(d)). Nevertheless, the EPO-mediated migration was dramatically decreased when BMSCs were further treated with AMD3100 (0.246 ± 0.029) as compared with the EPO group (*P* < 0.001). There was no significant difference between the AMD3100 group and the control group (*P* > 0.05).

### 3.3. EPO Promoted the Mobilization of GFP+ BMSCs In Vivo

**Figure 3:**
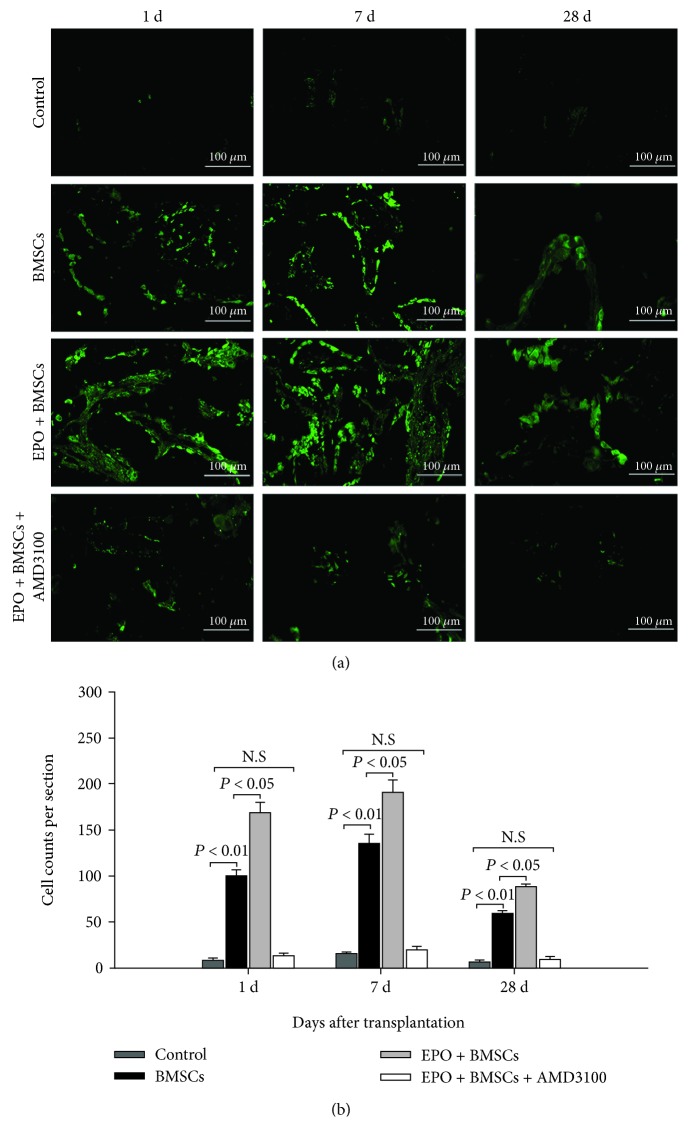
Immunofluorescent microscopy. (a) Immunofluorescence staining of GFP+ cells in four groups at each time point. BMSCs: bone marrow mesenchymal stem cells. Scale bar is 100 *μ*m. (b) Quantitative analysis of the number of GFP+ cells. The data are plotted as mean ± SD. N.S=no significant difference.

Fluorescent labeling of specimens in each group was analyzed at day 1, day 7, and day 28 after transplantation (Figures 3(a) and 3(b)). Cells were calculated in five randomly sorted fields, and the sum of cells for each section was determined. At day 1 and day 7, more GFP+ cells were observed in the EPO+BMSC group compared with the BMSC group (*P* < 0.05), and the number of GFP+ cells in the BMSCs group was significantly larger than that of the control group (*P* < 0.01). GFP+ cells reduced significantly at day 28, while the number of GFP+ cells was still greater in the EPO+BMSC group than that in the BMSC groups (*P* < 0.05). During the process of defect repair, only few GFP+ cells were observed in the control group. There was no significant difference between the EPO+BMSCs+AMD3100 and control groups (*P* > 0.05). The results indicated that blocking of the CXCR4 on BMSCs significantly reduced the migration of BMSCs to the defect areas.

### 3.4. Histological Analysis of Bone Defect Regeneration

**Figure 4:**
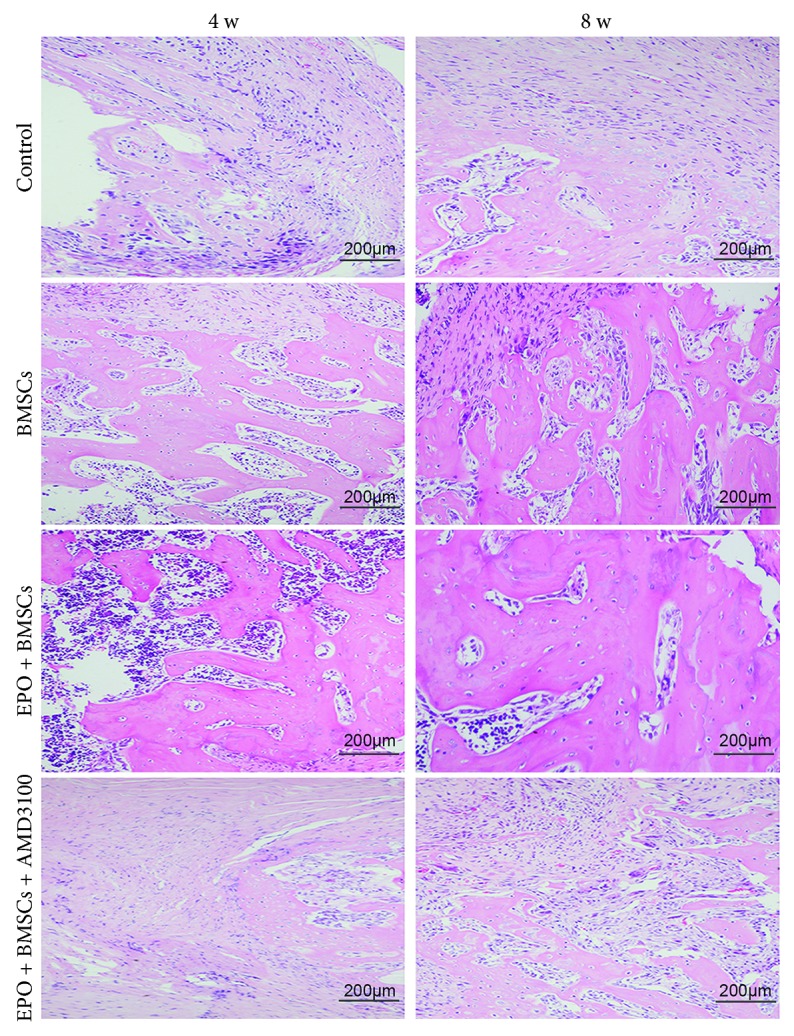
Histological examination of newly regenerated bone at 4 and 8 weeks after operation. Representative H&E images (scale bar is 200 *μ*m). New bone areas were stained in pink red.

**Figure 5:**
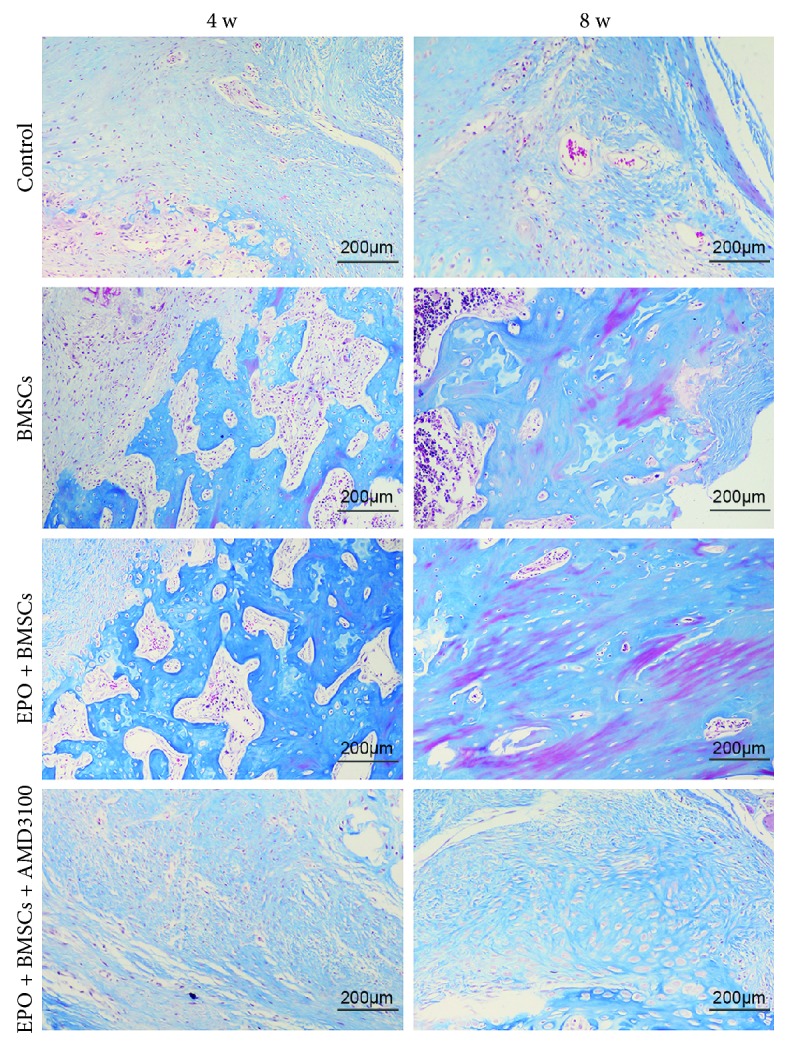
Representative Masson's trichrome staining images at 4 and 8 weeks after operation (scale bar is 200 *μ*m).

To further investigate how transplanted BMSCs recruited by EPO enhanced the bone regeneration, histologic analysis was conducted at 4 and 8 weeks after operation. Representative H&E images from each group and time point are shown in Figure 4 with newly generated bone stained in dark pink. At both the 4- and 8-week time points, robust bone growth among the defective areas was observed in the BMSC group and EPO+BMSC group. New blood vessels were also observed among the regenerated bone regions. However, the stained images of EPO+BMSCs showed more new bone formation and a more organized tissue structure than the BMSC alone group did. In contrast, the control group and the EPO+BMSCs+AMD3100 group showed minor new bone formation within the defective area along with fibrous connective tissue. Representative Masson's staining of new bone formation in each group was manifested in Figure 5. The regenerated bone in the BMSC group was less mature, with slightly red staining that indicates an earlier stage of mineralization. More new bones were formed in the EPO+BMSC group compared to the BMSC group. Furthermore, the novel bone in the EPO+BMSCs group was more mature, indicating a higher degree of mineralization. Few novel bones were observed in the control group and the EPO+BMSCs+AMD3100 group.

**Figure 6:**
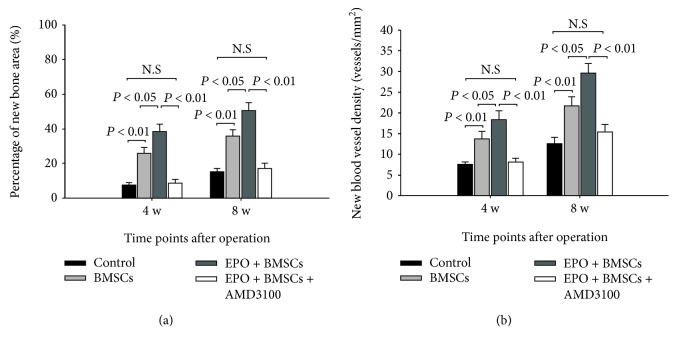
Histomorphometry analysis for bone defective model at 4 and 8 weeks. (a) Percentage of new bone area. (b) New blood vessel density. The data are plotted as mean ± SD. N.S = no significant difference.

To quantify the bone regenerative capacity of transplanted BMSCs, a new bone region was calculated via histomorphometry.  Figure 6(a) shows the percentage of new bone formation among the defected areas. The amount of novel bone was increased from 4 to 8 weeks in each group. The amount of new bone in the BMSC group was significantly higher than that of the control group (*P* < 0.01) at each time point. Also, more new bone formation was detected in the EPO+BMSC group compared to the BMSC group, with statistical significance (*P* < 0.05). The difference between the control group and the EPO+BMSC+AMD3100 group was not significant (*P* > 0.05). As shown in Figure 6(b), the EPO+BMSC group also had higher new vessel density at each implantation period than that of the BMSC group. There was no significant difference with regard to blood vessel density between the control and EPO+BMSC+AMD3100 groups at each time point (*P* > 0.05).

### 3.5. Mechanical Properties of the Bones

**Figure 7:**
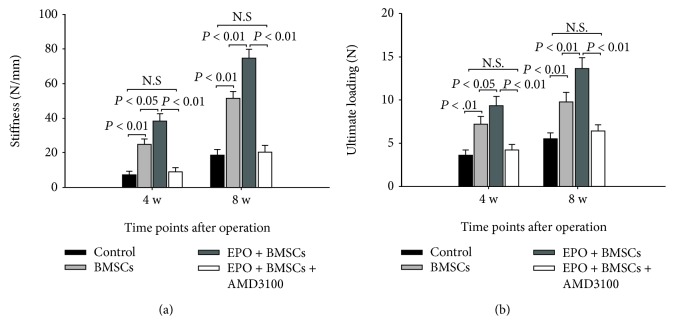
Three-point flexural test results at 4 and 8 weeks of postoperation. (a) The bending stiffness of tibia in each group. (b) The ultimate loading of tibia in each group. The data are plotted as mean ± SD. N.S = no significant difference.

The results of biomechanical testing are shown in Figures 7(a) and 7(b). At 4 weeks, the values of stiffness and ultimate loading in the EPO+BMSC group were significantly higher than those of the BMSC group (*P* < 0.05) and control group (*P* < 0.01), which is also significantly higher in the BMSC group than in the control group (*P* < 0.01). Nevertheless, no significant difference was found between the control and EPO+BMSC+AMD3100 groups (*P* > 0.05). The biomechanical findings at week 8 were similar to those at week 4, while the differences with regard to stiffness and ultimate loading of tibia between the EPO+BMSC and BMSC groups were more significant (*P* < 0.01). Together, these results indicate that combined transplantation of EPO and BMSCs increases mechanical strength of defected tibias.

### 3.6. Quantification of Bone Regeneration by Micro-CT

**Figure 8:**
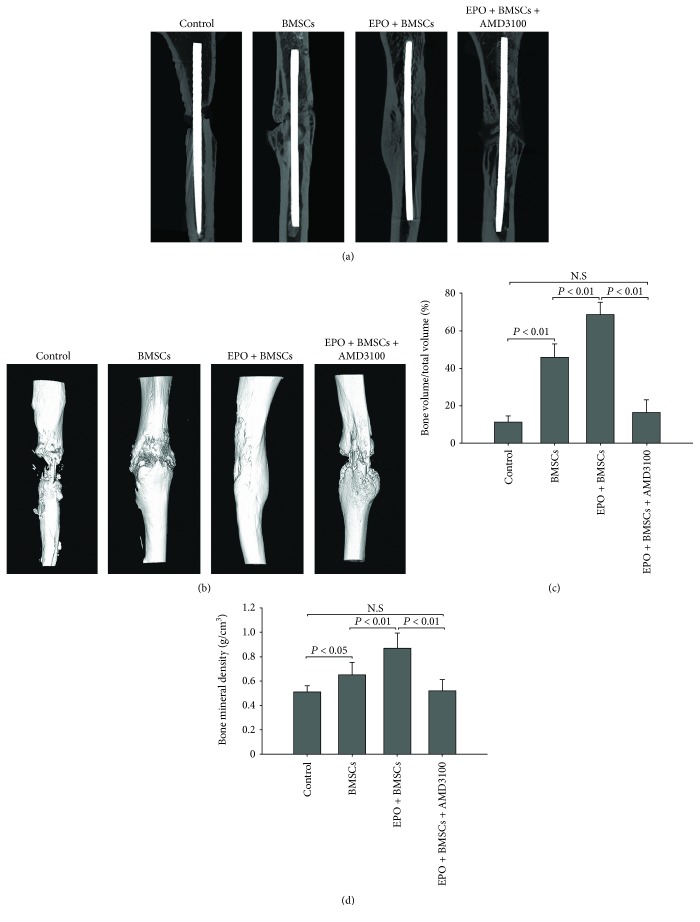
Micro-CT scans were used to evaluate in vivo new bone formation. (a, b) Representative 2D cross-sectional images and 3D reconstructed images at 8 weeks of postinjury. (c, d) Quantification of newly formed bone volume and bone mineral density by micro-CT. *N* = 6 for each population. The data are plotted as mean ± SD. N.S = no significant difference.

A long-term evaluation of bone volumetric restoration across the defective region was performed at the end of 8 weeks of postinjury. 2D cross-sectional images (Figure 8(a)) and 3D reconstructed images (Figure 8(b)) indicated that animals treated with EPO+BMSCs showed better bony bridging and callus formation compared to the BMSC alone group. There was little bone restoration in the control group. The quantification of regenerated bone volume (BV) and BMD of the regenerated bone confirmed the above findings. The mean values of BV/TV and BMD in the EPO+BMSC group were significantly greater (*P* < 0.01) than those of the BMSC alone group and control group (Figures 8(c) and 8(d)). Interestingly, there was no significant difference between the EPO+BMSC+AMD3100 and control groups (*P* > 0.05).

## 4. Discussion

Segmental bone defects arising from trauma, tumor, and congenital deformity have constituted challenging problems in orthopedic practice [2]. BMSCs have generated a great deal of excitement and potential in cell therapy applications, not only because of their ability to extensively self-renew and potentially differentiate into multiple cell groups but also for their lack of evident immunogenicity, which would enable allogeneic grafting without requirement for immunosuppressive drugs [26, 27]. Previous studies have already documented advantageous functions after systemic or localized administration of BMSCs for the restoration of injured cartilage [28], muscle [29], and bone [21]. Despite the potential of BMSCs in regenerative medicine, particularly for bone repairing, previous manipulation via BMSCs has shown serious limitations, such as compromised bone recruitment of transplanted cells, reduced proliferation and osteoblastic differentiation ability, and likely tumorigenesis [30, 31]. Therefore, a major objective of this study is to augment bone defect restoration by enhancing BMSC mobilization and homing, whereby cells are generated and integrated into the bone defect area, overcoming the aforementioned limitations.

Our current work demonstrates that BMSCs transplanted through the caudal vein can be mobilized by EPO to the bone defect area and participate in the regeneration of new bone. Based on histological analysis and micro-CT findings in this study, EPO has dramatic promotional effects on the osteogenesis and angiogenesis of BMSCs in vivo. Animals that have undergone EPO+BMSC transplant show a significant increase in new bone formation, organized tissue structure, new vessel density, callus formation, and BMD than the BMSC alone and control groups. At the biomechanical level, we demonstrated that combing transplantation of EPO and BMSCs enhances bone defect reconstruction by increasing the strength of the diaphysis, making it to be less fragile.

In recent decades, multiple investigators have proved that EPO is tissue-protective and anti-inflammatory and facilitates neurogenesis and angiogenesis [32]. Moreover, it has been established to have various biological functions, including rehabilitation of neuronal injury, promotion of the proliferation and differentiation ability of endothelial progenitor cells, and acceleration of wound healing [33]. It is well established that, while being discovered as a regulator of erythropoiesis, EPO is a significant growth factor that promotes the recruitment of BMSCs and subsequently triggers bone formation and angiogenesis from these BMSCs [34]. Chemokines and cytokines are significant factors in regulating mobilization, migration, and recruitment of stem cells [35]. Specifically, the SDF-1/CXCR4 axis has been reported to play a crucial role in the migration of BMSCs. Through Transwell assay, Wynn et al. [36] demonstrated the dose-related migration of human BMSCs to SDF-1, supporting the conclusion that the SDF-1/CXCR4 axis contributes to BMSC migration. In another study, with the help of the immobilized mouse tibia fracture model, Granero-Moltó et al. [37] proved the dynamic transferring of grafted BMSCs to the fracture region and their effects in the bone regeneration process, which is CXCR4 dose-dependent. On a similar note, the SDF-1/CXCR4 pathway was certified to play a significant role in the migration of BMSCs to sites of segmental bone defects which can facilitate endochondral bone repair. Interdiction of the SDF-1/CXCR4 pathway inhibited BMSCs transferring to the injured bone area, resulting in reduced callus formation [38]. Consistently, our in vitro and in vivo study (Figures 2 and 3) proved that BMSC engraftment could be mobilized by EPO to the bone defect area in a SDF-1/CXCR4-dependent manner, which was significantly blocked by AMD3100, the antagonist of CXCR4.

Observations of this study also indicated that engrafted BMSCs significantly increased new blood vessel density. Moreover, EPO infusion plus BMSC transplantation demonstrated a further increase in new vessel density compared with BMSCs alone. The high metabolic and low oxygen tension condition during bone regeneration increases tissue's demand for revascularization to deliver sufficient oxygen and nutrient and to clear up cellular debris [39]. Moreover, blood vessel density has been demonstrated to be positively correlated to bone formation rate. A temporal and spatial coupling of angiogenesis to bone regeneration and resorption has also been confirmed [40]. Hence, new blood vessels sprouting from existing bone tissue into the defect area are vital for osteogenesis. It has been demonstrated that EPO has angiogenesis capacity comparative to vascular endothelial growth factor (VEGF) and facilitates proliferation and migration of endothelial progenitor cells [41, 42]. These findings indicate that the beneficial effects of combined administration of EPO and BMSCs may be attributable, to a certain degree, to the angiogenic properties of EPO itself and EPO-mediated differentiation of BMSCs. Taken together, it is possible that EPO infusion and BMSC transplantation generate additive effects on new bone formation after injury.

One of the limitations in the current study is absence of the EPO alone control. While our study was focused on how EPO acts on exogenous BMSCs in the process of defective bone restoration and the intrinsic mechanism, EPO alone might also be involved in bone formation through direct and indirect pathways, which is worth investigating in the future.

## 5. Conclusions

Collectively, the present study indicates that BMSCs transplanted through the caudal vein can be mobilized by EPO to the bone defect area and participate in the new bone regeneration. The combined administration of EPO and BMSCs can achieve superior therapeutic osteogenesis and angiogenesis. The SDF-1/CXCR4 axis plays a key role in the EPO-induced mobilization and migration of BMSCs to the bone defect area. Therefore, combination therapy using EPO infusion and BMSC transplantation is potentially a novel therapeutic strategy for the reconstruction of segmented bones.

## Data Availability

The data used to support the findings of this study are available from the corresponding author upon request.

## Abbreviations

BMSCs: Bone marrow mesenchymal stem cells
EPO: Erythropoietin
BMD: Bone mineral density
CT: Computed tomography
SDF-1: Stromal cell-derived factor-1
CXCR4: CXC chemokine receptor 4
PBS: Phosphate-buffered saline
DMEM: Dulbecco's modified Eagle's medium
Ad-GFP: Adenovirus encoding green fluorescent protein
SD: Sprague-Dawley
DAPI: 4′,6-Diamidino-2-phenylindole
FBS: Fetal bovine serum
IBMX: 3-Isobutyl-1-methylxanthine
ECL: Enhanced chemiluminescence
ALP: Alkaline phosphatase
PVDF: Polyvinylidene fluoride
OCT: Optimal cutting temperature
SPSS: Statistical Package for the Social Sciences
ANOVA: Analysis of variance
OD: Optical density
BV: Bone volume
VEGF: Vascular endothelial growth factor.
